# Níveis Séricos do BDNF na Proteção Cardiovascular e em Resposta ao Exercício

**DOI:** 10.36660/abc.20190368

**Published:** 2020-08-19

**Authors:** Ivani Credidio Trombetta, José Roberto DeMoura, Cleber Rene Alves, Renato Carbonari-Brito, Felipe Xerez Cepeda, José Ribeiro Lemos

**Affiliations:** 1 Universidade Nove de Julho São Paulo SP Brasil Universidade Nove de Julho (UNINOVE), São Paulo, SP - Brasil; 2 Escola de Educação Física Polícia Militar do Estado de São Paulo São Paulo SP Brasil Escola de Educação Física da Polícia Militar do Estado de São Paulo, São Paulo, SP – Brasil

**Keywords:** Doenças Cardiovasculares/mortalidade, BDNF, Fator Neurotrófico Derivado do Encéfalo, Endotélio Vascular, Fatores de Crescimento Neural, Plasticidade Neuronal, Polimorfismo, Exercício Físico

## Abstract

As doenças cardiovasculares (DCV) são atualmente a maior causa de morte no Brasil e no mundo. Em 2016 as DCV foram responsáveis por mais de 17 milhões de mortes, representando 31% de todas as mortes em nível global. Mecanismos moleculares e genéticos podem estar envolvidos na proteção cardiovascular e devem ser considerados nas novas abordagens terapêuticas. Nesse sentido, recentes estudos têm relatado que o Fator Neurotrófico Derivado do Encéfalo (Brain-Derived Neurotrophic Factor, BDNF) está reduzido em indivíduos predispostos a desenvolverem DCV, e que o treinamento físico aeróbio aumenta as quantidades de BDNF circulante. O BDNF é uma neurotrofina encontrada em altas concentrações no hipocampo e córtex cerebral, sendo considerada molécula-chave na manutenção da plasticidade sináptica e na sobrevivência das células neuronais. Além da plasticidade neuronal, BDNF também é importante na função vascular, promovendo angiogênese por meio da regulação por espécies reativas de oxigênio (ROS). Entretanto, uma variante do gene do BDNF em humanos, o polimorfismo Val66Met (substituição do aminoácido valina por uma metionina na posição 66 do códon), que ocorre em 20-30% da população caucasiana, pode afetar as concentrações de BDNF no plasma e sua atividade em todos os tecidos periféricos contendo receptores tirosina quinase B (TrkB), como o endotélio. De fato, recentemente observamos que o polimorfismo Val66Met prejudica a reatividade vascular e o BDNF circulante em resposta ao treinamento físico. Dessa forma, apresentaremos a seguir uma discussão sobre os níveis séricos de BDNF na proteção cardiovascular, a variante genética Val66Met na reatividade vascular e o efeito do exercício físico.

## Introdução

As principais causas de morte por doenças não transmissíveis são por doenças cardiovasculares (DCV). Mundialmente, as mortes por DCV aumentaram 12,5% entre 2005 e 2015, chegando a 17,9 milhões de mortes.^1^ No Brasil, a mortalidade por DCV representou 28% do total de óbitos ocorridos nos últimos cinco anos, atingindo 38% dos óbitos na faixa etária produtiva (18 a 65 anos).^2^

As DCV mais relevantes em termos de saúde pública são as doenças cardíacas (doença arterial coronariana e insuficiência cardíaca) e cerebrovasculares. Os fatores de risco para DCV são bem conhecidos (entre eles, obesidade, dislipidemia, diabetes e sedentarismo). No entanto, a sua base molecular é complexa e está ligada a uma ampla gama de vias biológicas, incluindo o metabolismo de lipídios e glicose, inflamação, reparo vascular e angiogênese.

A etiologia principal das DCV é a aterosclerose, um complexo processo inflamatório crônico da parede arterial que envolve o recrutamento e ativação de células na lesão intimal. Essa ativação de células endoteliais por citocinas inflamatórias e lipoproteínas oxidadas, seguida pelo aumento da adesão de monócitos circulantes no sangue ao endotélio e a migração de células musculares lisas vasculares para a camada neo-íntima em desenvolvimento, leva ao desenvolvimento da placa aterosclerótica, obstruindo progressivamente o lúmen vascular e reduzindo o fluxo sanguíneo.^3^ Adicionalmente, na aterosclerose ocorre a disfunção endotelial, caracterizada pela redução da biodisponibilidade de óxido nítrico (NO) na parede dos vasos sanguíneos.^4^

A disfunção endotelial é um marcador de risco cardiovascular e está presente nas DCV, como hipertensão arterial, doença arterial coronariana e insuficiência cardíaca crônica.^5^ Diversos fatores têm sido associados com a modulação do fluxo sanguíneo endotélio dependente, tais como a biodisponibilidade de L-arginina, de tetraidrobiopterina (BH4), índices de LDL-colesterol e o fator de crescimento endotelial vascular (VEGF), entre outros.^4^

Embora a proteína fator neurotrófico derivado do encéfalo (BDNF) esteja diretamente relacionada com a saúde dos neurônios,^6^ estudos experimentais translacionais e clínicos vêm demonstrando sua forte relação com o sistema vascular. De fato, inicialmente as neurotrofinas tiveram suas ações identificadas basicamente no desenvolvimento e amadurecimento do sistema nervoso. No entanto, desde o final dos anos 1990, surgiram fortes evidências na literatura que as neurotrofinas estão implicadas em importantes funções cardiovasculares.^7^ Mais recentemente, um importante estudo demonstrou a relação do BDNF circulante com o sistema vascular, especificamente com a angiogênese, por meio da regulação por espécies reativas de oxigênio (ROS).^8^ Portanto, além da função no sistema nervoso, evidências acumuladas sugerem que o BDNF também é importante para o sistema cardiovascular.

Devido à relação do BDNF com a angiogênese, com o aumento da vasodilatação e perfusão tecidual, ele é mais um elo importante entre estilo de vida e saúde vascular, com repercussões na estrutura cerebral e função cognitiva em adultos idosos.^9^ Um estilo de vida que inclua engajamento cognitivo, prática regular de exercício físico e dieta saudável é estratégia chave para manter a saúde cerebral durante o envelhecimento.^9^

Nesse contexto, vários estudos demonstraram que o exercício é um dos principais fatores no aumento dos níveis séricos de BDNF^10 - 12^ e que o aumento dos níveis do BDNF é o elemento chave que liga o exercício aos benefícios cognitivos.^13^ Entretanto, as variações na concentração de BDNF circulante, inclusive seu aumento em resposta ao treinamento físico,^12^ podem ser explicadas por uma variante genética do BDNF, um polimorfismo funcional de nucleotídeo único (SNP), responsável pela substituição do aminoácido Valina por uma Metionina na posição 66 do códon. O polimorfismo Val66Met, condição que ocorre em 20-30% da população caucasiana,^14 - 16^ prejudica a secreção regulada e o tráfego intracelular de BDNF.^14 , 17^ Estas novas descobertas têm aberto um novo campo de investigação em medicina cardiovascular e terapêutica.

### Fator Neurotrófico Derivado do Encéfalo (BDNF)

O BDNF é a neurotrofina mais expressa no sistema nervoso central, encontrada em altas concentrações no hipocampo e córtex cerebral. É molécula-chave envolvida na manutenção da plasticidade sináptica e na sinaptogênese do hipocampo, local de aquisição e consolidação da memória.^18 , 19^ A produção e secreção alteradas de BDNF foram demonstradas em várias doenças neurodegenerativas, como Alzheimer e Parkinson.^20 - 22^ Em indivíduos cognitivamente normais, a concentração de BDNF no líquido cefalorraquidiano diminui ao longo da vida na ausência de demência, sendo que a menor concentração de BDNF no líquido cefalorraquidiano foi associada fortemente com memória prejudicada e menor função executiva.^23^ Conhecimentos atuais apontam para o fato de que a cognição anormal está associada à diminuição BDNF no hipocampo, sendo esse um fator determinante do prejuízo de fatores como aprendizagem, depressão, humor, transtornos de ansiedade e esquizofrenia.^24^

Enquanto o BDNF promove sobrevivência neuronal e realça a plasticidade sináptica pela ativação de seu receptor tirosina-quinase B (TrkB), seu predecessor pró-BDNF atua de maneira antagônica, resultando em apoptose celular ao interagir com o receptor p75 das neurotrofinas (p75NTR). Essa importante função demonstra que ambos estão envolvidos em diferentes funções fisiológicas.^25 , 26^

O BDNF é produzido pré-sinapticamente nos corpos celulares dos neurônios sensoriais projetados no corno dorsal, enquanto que no hipocampo é produzido predominantemente pelos dendritos pós-sinápticos.^22 , 27 , 28^ Perifericamente, o BDNF sérico é encontrado nas plaquetas do plasma sanguíneo e é formado pelas células do endotélio vascular e pelas células sanguíneas mononucleadas periféricas.^29 , 30^ Seu potencial terapêutico é caracterizado por sua capacidade de atravessar livremente a barreira hemato-encefálica em ambas as direções, via alta capacidade de saturação do sistema transportador.^22 , 30 , 31^ No sistema nervoso periférico, o BDNF ainda apresenta um papel adicional, atuando na regeneração axonal. Vale ressaltar que o gene BDNF e seu receptor TrkB são expressos não só no encéfalo, mas também em outros locais do organismo, tais como coração, pulmões e tecido endotelial,^26 , 32 , 33^ demonstrando sua funcionalidade em outros órgãos e tecidos do organismo.

O gene BDNF está localizado no braço curto (p) do cromossomo 11 (11p13) e compreende 11 exons e 9 promotores funcionais.^34^

Um polimorfismo funcional de ocorrência natural no gene do BDNF humano no nucleotídeo 196 (G/A) codifica uma substituição de aminoácido valina pela metionina na posição 66 (Val66Met ou Met66Met). Este polimorfismo resulta em menor produção e menores quantidades circulantes de BDNF^14^ e tem sido associado a maior suscetibilidade de distúrbios neurodegenerativos. Funcionalmente, os polimorfismos Met66Met e Val66Met causam prejuízos no tráfego intracelular e na secreção regulada em neurônios.^14 , 17^

De fato, a herança desse polimorfismo tem sido associada com pior desempenho cognitivo em idosos saudáveis^35^ e prejudica a memória de indivíduos.^14^ Além disso, o polimorfismo Val66Met leva a um volume hipocampal 4 a 11% menor, observado por ressonância magnética em adultos saudáveis.^23^

### BNDF e Função Cardiovascular

A ligação entre as doenças cardíacas e deterioração cognitiva tem sido reportada na literatura.^36 , 37^ Alguns autores acreditam que o mecanismo da “demência cardiogênica” envolva a hipoperfusão cerebral crônica devido à redução no débito cardíaco por várias doenças cardiovasculares.^38 , 39^ Embora a associação entre distúrbios cognitivos e fatores de risco cardiovascular seja complexa e possivelmente mediada por diferentes mecanismos, a presença de alterações microvasculares cerebrais, clinicamente manifestas ou silenciosas, estão envolvidas. Adicionalmente, recente estudo^24^ forneceu novos insights sobre o potencial mecanismo molecular pelo qual a doença cardíaca induza a disfunção cerebral. Esses autores, estudando um modelo de camundongo transgênico que tem superexpressão cardíaca específica de microRNA-1-2 (miR-1-2), observaram que a superexpressão cardíaca do miR-1 também induziu a anormalidades comportamentais que estão associadas à regulação negativa da expressão do BDNF no hipocampo. A compreensão mais ampla da forma pelo qual as doenças cardíacas afetam a função cognitiva pode levar a novas estratégias terapêuticas.

A importância dos níveis circulantes do BDNF na proteção cardiovascular ficou evidente no estudo prospectivo de coorte do *Framingham Heart Study* (FHS).^40^ Para avaliar uma relação potencialmente causal entre os níveis de BDNF e DCV, foi realizada uma análise de randomização mendeliana usando as metas do exemplo CARDIoGRAM (Gene de Doença da Artéria Coronária Genômica-Replicação Ampla e Meta-Análise). Nesse estudo, realizado com uma grande amostra de base comunitária, os investigadores observaram que níveis mais elevados de BDNF estão associados a menor risco de eventos cardiovasculares e morte, independente dos fatores de risco padrão, incluindo marcadores de inflamação de baixo grau, índice de massa corporal (IMC), atividade física e depressão.^40^

De fato, um papel importante do BDNF no sistema cardiovascular é a promoção da angiogênese vascular e aumento na densidade capilar.^41^ Estudos revelaram que o BDNF atua nas células endoteliais promovendo a neovascularização em resposta a estímulos hipóxicos por meio da via de Akt.^42 - 44^

A primeira evidência do envolvimento do BDNF no processo de angiogênese veio do estudo de Donovan et al.,^45^ sobre o desenvolvimento do miocárdio embrionário, no qual foi evidenciado que a superexpressão do BDNF está associada a um aumento da densidade capilar. Recentemente, um elegante estudo experimental demonstrou, pela primeira vez, que o BDNF promove a formação de tubos angiogênicos por meio da geração de ROS derivados da NADPH oxidase (NOX) pela transdução de sinal do receptor TrkB, provavelmente via ativação de Akt, resultando na migração de células endoteliais.^8^ O estudo sugere que: TrkB ⇒ NADPH oxidase 2 (Nox2) ⇒ ROS ⇒ Phosphoinositide 3-kinase (PI3K)/Akt.^8^

De fato, o BDNF tem sido consistentemente implicado na angiogênese e na manutenção da integridade vascular. Especificamente no endotélio, além da ligação do BDNF com seu receptor de alta afinidade TrkB^25 , 46^ há, também, a expressão do receptor p75, cuja ligação com o pró-BDNF tem sido relacionada com apoptose de músculos lisos vasculares.^47 , 48^ Considerando a localização conjugada do BDNF-TrkB e pró-BDNF-p75 no endotélio e devido à atuação fisiológica antagônica existente entre o BDNF e pró-BDNF, é importante se levar em conta o equilíbrio entre plasticidade/sobrevivência e apoptose sobre o fluxo sanguíneo periférico através da razão BDNF/pró-BDNF.

Mais recentemente, a ligação entre esse neurotrófico e a proteção cardiovascular foi evidenciada em estudo de Okada et al.,^49^ realizado com camundongos knockout condicionais de BDNF, nos quais a expressão do BDNF foi sistemicamente reduzida. Nesse estudo os autores demonstraram que um mecanismo mediado pelo Sistema Nervoso Central está envolvido na regulação da função cardíaca após o infarto do miocárdio. Os insultos isquêmicos são transmitidos do coração para o Sistema Nervoso Central através de fibras nervosas aferentes cardíacas após o infarto do miocárdio, aumentando assim a expressão neuronal do BDNF. Um aumento no BDNF circulante promove a sobrevivência dos cardiomiócitos e está associado ao aumento da expressão de fatores pró-angiogênicos. Comparativamente, os animais knockout tiveram maior dano miocárdico após o infarto experimental em comparação com camundongos do tipo selvagem.^49^

Neste contexto, o polimorfismo Val66Met pode afetar as concentrações séricas de BDNF e, consequentemente, influenciar a atividade dos tecidos que contenham os receptores TrkB, sejam eles neurônios ou mesmo tecidos periféricos, como as células endoteliais vasculares.

### BDNF e Efeitos Cognitivos do Exercício

Há muitas evidências de que o exercício físico, principalmente o aeróbio, tem efeito benéfico em domínios cognitivos, particularmente nas funções executivas e de memória e reduz a atrofia hipocampal no final da idade adulta, e que o BDNF está fortemente envolvido.^11 , 50 - 57^

Estudos epidemiológicos e de intervenção reforçam a ideia de usar a atividade física como uma estratégia para aumentar a neuroplasticidade em condições patológicas.^58^ Vários estudos têm demonstrado que o exercício físico não apenas causa mudanças estruturais no cérebro, mas também protege contra o declínio cognitivo relacionado ao envelhecimento.^57 , 59^

O exercício físico ativa cascatas moleculares e celulares que promovem a plasticidade neuronal e a neurogênese, induzindo a expressão do gene que codifica o BDNF.^10 , 60^ As concentrações periféricas de BDNF aumentam tanto no exercício aeróbico agudo como no crônico, sendo que a magnitude desse aumento parece ser dependente da intensidade do exercício.^61^

Adicionalmente, maiores benefícios cognitivos são obtidos quanto maior for a duração do programa e da sessão de exercícios e quanto mais idosos forem os indivíduos, sendo que as mulheres apresentam maiores benefícios que os homens.^56^ A diferença entre os gêneros no BDNF do líquido cefalorraquidiano a favor das mulheres pode ser devido a efeitos hormonais,^23^ já que os receptores estrogênicos se localizam nas células que expressam o BDNF e seu receptor TrkB, sendo que o estrogênio regula a expressão de BDNF.^62^

Interessantemente, esse benefício do exercício ocorre mesmo em homens adultos jovens. Isso foi evidenciado num estudo de coorte com homens jovens suecos alistados no serviço militar aos 18 anos (n=1.221.727),^50^ no qual foi encontrada significativa associação positiva entre a aptidão cardiovascular e desempenho cognitivo após o ajuste para fatores de confusão relevantes.

Em grande parte, os benefícios do exercício sobre a produção do BDNF e a plasticidade neuronal estão relacionados ao aumento da vascularização cerebral e muscular. De fato, em uma recente revisão^63^ os autores evidenciaram que os benefícios cognitivos decorrentes da boa aptidão cardiovascular estão relacionados ao aumento da circulação cerebral e à angiogênese. Essa importante adaptação permite o aumento do fluxo e a regulação positiva das neurotrofinas no nicho neurogênico do hipocampo, fenômeno que ocorre mesmo após as sessões agudas de exercícios.^63^

Especificamente, estudos sobre o efeito do exercício agudo e crônico sobre a concentração sérica de BDNF ainda trazem resultados controversos. Por exemplo, em estudo comparando os efeitos crônicos e agudos do exercício físico sobre a concentração sérica de BDNF, ficou demonstrado que uma única sessão de exercício foi capaz induzir aumento transitório nos níveis de BDNF, porém os mesmos resultados não foram alcançados em um período maior de treinamento.^64^ Em contrapartida, em outro estudo onde a amostra foi submetida a 6 meses de treinamento, foi encontrada tendência de aumento na concentração sérica de BDNF, além de melhora na função cognitiva.^65^ Resultado semelhante foi encontrado em um estudo longitudinal com idosos, que apresentou como resultado aumento no volume de partes do hipocampo e, de acordo com os autores, esse fato está relacionado com o aumento nos níveis de BDNF.^51^

Esses resultados aparentemente controversos podem ser dependentes do curso temporal dos benefícios do exercício especificamente nos níveis plasmáticos de BDNF após exercício, isto é, se logo após uma única sessão de exercício agudo, se após uma sessão após um programa de exercício regular (mostrando alterações na liberação de BDNF depois de repetidas sessões de exercício) ou alterações nos níveis de BDNF no repouso após um programa de exercícios regulares.^66^ De fato, isso foi evidenciado na recente meta-análise sobre os efeitos do exercício no BDNF sérico,^66^ que concluiu que o exercício regular intensificou o efeito de uma sessão de exercício nos níveis de BDNF (g de Hedges=0,59; *P* =0,02). Porém, os resultados indicaram um menor efeito do exercício regular nos níveis de BDNF em repouso (g de Hedges=0,27; *P* =0,005). Existem evidências confiáveis de estudos em humanos indicando que cada episódio de exercício resulta em uma dose-resposta de BDNF e que a magnitude dessa resposta pode ser aumentada ao longo do tempo através de exercícios regulares.^66^

Há grande corpo de evidências que demonstra que o exercício atua em diversas e poderosas vias neuroprotetoras, que podem convergir para promover a saúde cerebral continuada até a velhice. Esses benefícios ocorrem seja em resposta a atividades agudas, seja na prática regular, e ocorre tanto em resposta aos exercícios de alta intensidade como em exercícios aeróbios de intensidade moderada, aumentando os níveis de fatores neurotróficos circulantes e a neurotransmissão, exercendo efeitos benéficos sobre o humor e funções cognitivas em indivíduos de todas as idades.

### BDNF e Efeitos Cardiovasculares do Exercício

No sistema cardiovascular o BDNF pode estar envolvido, pelo menos em parte, nos benefícios endoteliais vasculares. Além disso, estudo recente evidenciou que homens idosos ativos apresentam níveis plasmáticos de BDNF significativamente mais altos quando comparados aos seus pares inativos. Nesse estudo o BDNF se correlacionou com o VO_2_máx (R=0,765; p<0,001). Adicionalmente, houve correlação inversa entre o BDNF e o índice aterogênico (TC/HDL), hsCRP e oxLDL. Esses achados demonstram que um alto nível de aptidão cardiorrespiratória está associado a um nível mais alto de BDNF circulante, que por sua vez está relacionado à menor risco cardiovascular.^67^

É possível que os polimorfismos possam influenciar os efeitos benéficos do exercício. Recentemente, observamos que a reatividade vascular periférica e as respostas séricas de BDNF ao treinamento físico estão prejudicadas pelo polimorfismo Val66Met do BDNF, responsividade esta que está associada às concentrações séricas de BDNF em indivíduos saudáveis.^12^

**Figura 1:**
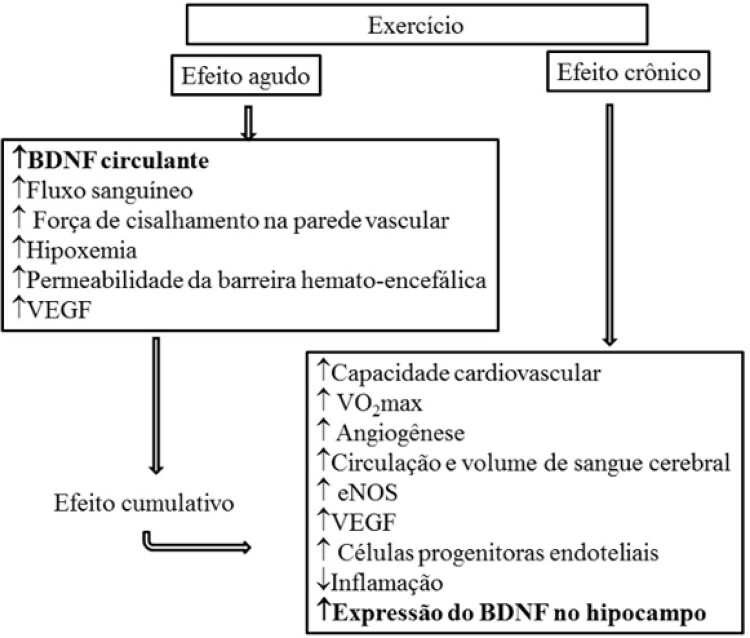
**–** Efeito agudo e crônico do exercício físico sobre os aspectos cardiovasculares relacionados com o BDNF (Adaptado de Stimpson et al, 2018).

Considerando todo o exposto, a importância do exercício físico em promover a saúde do cérebro e cardiovascular está ganhando reconhecimento, seja na condição fisiológica do envelhecimento cerebral, seja em indivíduos afetados pelos estágios iniciais da neurodegeneração. De fato, os vários estudos em animais e em humanos sugerem que a atividade física pode reduzir o risco de declínio cognitivo e, portanto, um estilo de vida ativo pode ser considerado uma estratégia preventiva da deterioração da saúde cerebral, assim como ocorre com a disfunção cardiovascular.

Sem dúvida, com o aumento da longevidade, abordagens preventivas de longo-prazo, com ênfase na promoção de hábitos positivos de saúde que atrasem a progressão e o declínio cognitivo, são cada vez mais importantes. Vale lembrar que além de modular o ambiente interno do cérebro, a prática regular de exercício físico atua diretamente sobre o sistema cardiovascular, imunológico e metabólico, tendo um papel essencial em um estilo de vida saudável.
