# Supplementary material for: Níveis Séricos do BDNF na Proteção Cardiovascular e em Resposta ao Exercício
Source: Arq Bras Cardiol. 2020 Aug 19;115(2):263–9. [Article in Portuguese] doi: 10.36660/abc.20190368 (PMC8384297; doi:10.36660/abc.20190368)
Supplement: Translation

# Translation: Serum Levels of BDNF in Cardiovascular Protection and in Response to Exercise

**Authors:** Ivani Credidio Trombetta, José Roberto DeMoura, Cleber Rene Alves, Renato Carbonari-Brito, Felipe Xerez Cepeda, José Ribeiro Lemos

**Affiliations:** 1 Brazil Universidade Nove de Julho (UNINOVE), São Paulo, SP - Brazil; 2 Brazil Escola de Educação Física da Polícia Militar do Estado de São Paulo, São Paulo, SP – Brazil

**Keywords:** Cardiovascular Diseases/mortality, BDNF, Brain-Derived Neurotrophic Factor, Endothelium Vascular, Nerve Growth Factors, Neuronal Plasticity, Polymorphism, Exercise

## Abstract

Cardiovascular disease (CVD) is currently the leading cause of death in Brazil and worldwide. In 2016, CVD accounted for more than 17 million deaths, representing 31% of all deaths globally. Molecular and genetic mechanisms may be involved in vascular protection and should be considered in new therapeutic approaches. In this sense, recent studies have reported that brain-derived neurotrophic factor (BDNF) is reduced in individuals predisposed to develop CVD, and that aerobic physical training increases the amounts of circulating BDNF. BDNF is a neurotrophin found at high concentrations in the hippocampus and cerebral cortex and is considered a key molecule for the maintenance of synaptic plasticity and survival of neuronal cells. In addition to neuronal plasticity, BDNF is also important in vascular function, promoting angiogenesis through the regulation of reactive oxygen species (ROS). However, a variant of the BDNF gene in humans, the Val66Met polymorphism (substitution of the amino acid valine for a methionine at position 66 of the codon), occurring in 20-30% of the Caucasian population, may affect plasma BDNF concentrations and its activity in all peripheral tissues containing tyrosine kinase B receptors (TrkB), such as the endothelium. Thus, we will present a discussion about the role of serum BDNF levels in cardiovascular protection, Val66Met genetic variant in vascular reactivity and the effect of physical exercise.

## Introduction

The main causes of death from noncommunicable diseases are cardiovascular diseases (CVD). Across the world, CVD deaths increased 12.5% between 2005 and 2015, reaching 17.9 million deaths.^1^ In Brazil, CVD mortality accounted for 28% of all deaths in the last five years, accounting for 38% of all deaths in the productive age range (18 to 65 years).^2^

The most relevant CVD in terms of public health are heart (coronary artery disease and heart failure) and cerebrovascular diseases. Risk factors for CVD are well known (among them, obesity, dyslipidemia, diabetes and sedentary lifestyle). However, its molecular basis is complex and is linked to a wide range of biological pathways, including lipid and glucose metabolism, inflammation, vascular repair and angiogenesis.

The main etiology of CVD is atherosclerosis, a complex chronic inflammatory process of the arterial wall that involves the recruitment and activation of cells in the lesion of the intima layer. This endothelial cell activation by inflammatory cytokines and oxidized lipoproteins, followed by increased adhesion of circulating blood monocytes to the endothelium and migration of vascular smooth muscle cells into the developing neointimal layer, leads to the development of the atherosclerotic plaque, progressively obstructing the vascular lumen and reducing blood flow.^3^ In addition, atherosclerosis occurs in endothelial dysfunction, characterized by reduced bioavailability of nitric oxide (NO) on the wall of blood vessels.^4^

Endothelial dysfunction is a marker of cardiovascular risk and is present in CVD such as hypertension, coronary artery disease and chronic heart failure.^5^ Several factors have been associated with the endothelium-dependent blood flow modulation, such as the bioavailability of L-arginine, tetrahydrobiopterin (BH4), LDL-cholesterol and vascular endothelial growth factor (VEGF) levels, among others.^4^

Although the brain-derived neurotrophic factor (BDNF) is directly related to the health of neurons,^6^ translational and clinical experimental studies have demonstrated their strong association with the vascular system. In fact, initially neurotrophins had their actions identified basically in the development and maturation of the nervous system. However, since the late 1990s, strong evidence has emerged in the literature that neurotrophins are implicated in important cardiovascular functions.^7^ More recently, an important study has demonstrated the association of circulating BDNF to the vascular system, specifically angiogenesis, through the regulation of reactive oxygen species (ROS).^8^ Thus, in addition to the nervous system function, accumulated evidence suggests that BDNF is also important for the cardiovascular system.

Because of the association between BDNF and angiogenesis, increased vasodilation and tissue perfusion, this neurotrophin is another important link between lifestyle and vascular health, with repercussions on brain structure and cognitive function in older adults.^9^ A lifestyle that includes cognitive engagement, regular exercise, and a healthy diet is a key strategy to maintain brain health during the aging process.^9^

In this context, several studies have shown that exercise is one of the main factors in increasing serum BDNF levels^10 - 12^ and that increasing BDNF levels is the key element that links exercise to cognitive benefits.^13^ However, variations in the levels of circulating BDNF, including its increase in response to physical training,^12^ can be explained by a genetic variant of BDNF, a functional single-nucleotide polymorphism (SNP), responsible for the substitution of the amino acid Valine to Methionine at position 66 of the codon. The Val66Met polymorphism, a condition that occurs in 20-30% of the Caucasian population,^14 - 16^ impairs both regulated secretion and intracellular traffic of BDNF.^14 , 17^ These new findings have opened a new field of research in cardiovascular and therapeutic medicine.

### Brain-Derived Neurotrophic Factor (BDNF)

BDNF is the most expressed neurotrophin in the central nervous system, found at high concentrations in the hippocampus and cerebral cortex. It is a key molecule involved in the maintenance of synaptic plasticity and synaptogenesis of the hippocampus, a site of memory acquisition and consolidation.^18 , 19^ The altered production and secretion of BDNF have been demonstrated in several neurodegenerative disorders , such as Alzheimer’s and Parkinson’s disease.^20 - 22^ In cognitively normal individuals, the concentration of BDNF in the cerebrospinal fluid decreases throughout life in the absence of dementia, and a lower concentration of BDNF in the cerebrospinal fluid was strongly associated with impaired memory and lower executive function.^23^ Current knowledge points to the fact that abnormal cognition is associated with BDNF decrease in the hippocampus, which is a determining factor in the impairment of factors such as learning skills, depression, mood, anxiety disorders and schizophrenia.^24^

While BDNF promotes neuronal survival and enhances synaptic plasticity by activating its tyrosine kinase receptor B (TrkB), its precursor, proBDNF, acts antagonistically, resulting in cell apoptosis when interacting with the p75 receptor of neurotrophins (p75NTR). This important function demonstrates that both are involved in different physiological functions.^25 , 26^

The BDNF is produced presynaptically in the cell bodies of the sensory neurons projected in the dorsal horn, whereas in the hippocampus it is produced predominantly by the postsynaptic dendrites.^22 , 27 , 28^ Peripherally, serum BDNF is found in blood plasma platelets and consists of vascular endothelial cells and peripheral mononuclear blood cells.^29 , 30^ Its therapeutic potential is characterized by its ability to freely cross the blood-brain barrier in both directions via high saturation capacity of the carrier system.^22 , 30 , 31^ In the peripheral nervous system, BDNF still plays an additional role, acting on axonal regeneration. It is worth mentioning that the BDNF gene and its TrkB receptor are expressed not only in the brain, but also in other parts of the body, such as the heart, lungs and endothelial tissue,^26 , 32 , 33^ demonstrating its function in other organs and tissues of the body.

The BDNF gene is located on the short arm (p) of chromosome 11 (11p13) and comprises 11 exons and 9 functional promoters.^34^

A naturally-occurring functional polymorphism in the human BDNF gene at nucleotide 196 (G/A) encodes a substitution of amino acid valine to methionine at position 66 (Val66Met or Met66Met), which besides resulting in lower production and circulating amounts of BDNF,^14^ has been associated with greater susceptibility to neurodegenerative disorders. Functionally, the Met66Met and Val66Met polymorphisms cause impairments in the intracellular traffic and in regulated secretion in neurons.^14 , 17^

In fact, the inheritance of this polymorphism has been associated with poor cognitive performance in healthy elderly individuals^35^ and memory impairment of individuals.^14^ Additionally, the Val66Met polymorphism leads to 4 to 11% lower hippocampal volume observed by magnetic resonance imaging in healthy adults.^23^

### BNDF and Cardiovascular Function

The link between heart disease and cognitive impairment has been reported in the literature.^36 , 37^ Some authors believe that the mechanism of “cardiogenic dementia” involves chronic cerebral hypoperfusion caused by the reduction in cardiac output due to various cardiovascular diseases.^38 , 39^ Although the association between cognitive disorders and cardiovascular risk factors is a complex one and possibly mediated by different mechanisms, the presence of clinically manifest or silent cerebral microvascular changes are involved. In addition, a recent study^24^ provided new insights into the potential molecular mechanism by which heart disease induces brain dysfunction. These authors, studying a transgenic mouse model that has specific microRNA-1-2 (miR-1-2) cardiac overexpression, have observed that cardiac overexpression of miR-1 also induced behavioral abnormalities that are associated with the negative regulation of BDNF expression in the hippocampus. A broader understanding of how heart disease affects cognitive function may lead to new therapeutic strategies.

The importance of circulating levels of BDNF in cardiovascular protection was evident in the prospective cohort study of the Framingham Heart Study (FHS).^40^ To evaluate a potentially causal association between the levels of BDNF and CVD, a Mendelian randomization analysis was performed using the goals of the CARDIoGRAM (Coronary Artery Disease Genome-Wide Replication and Meta-Analysis) study. In this study, conducted with a large community-based sample, the researchers observed that higher levels of BDNF are associated with a lower risk of cardiovascular events and death, regardless of the standard risk factors, including low-grade inflammation markers, body mass index (BMI), physical activity and depression.^40^

In fact, an important role of BDNF in the cardiovascular system is the promotion of vascular angiogenesis and increase in capillary density.^41^ Studies have shown that BDNF acts on endothelial cells promoting neovascularization in response to hypoxic stimuli via the Akt pathway.^42 - 44^

The first evidence of BDNF involvement in the angiogenesis process came from the study by Donovan et al.^45^ about the development of the embryonic myocardium, in which it was shown that the overexpression of BDNF is associated with an increase in capillary density. Recently, an elegant experimental study demonstrated for the first time that BDNF promotes the formation of angiogenic tubes through the generation of ROS derived from NADPH oxidase (NOX) by TrkB receptor signal transduction, probably via Akt activation , resulting in the migration of endothelial cells.^8^ The study suggests that: TrkB ⇒ NADPH oxidase 2 (Nox2) ⇒ ROS ⇒ Phosphoinositide 3-kinase (PI3K)/Akt.^8^

In fact, BDNF has been consistently implicated in the angiogenesis and maintenance of vascular integrity. Specifically in the endothelium, besides the binding of BDNF to its high affinity receptor TrkB,^25 , 46^ there is also the expression of the p75 receptor, of which binding to the pro-BDNF has been related to vascular smooth muscle apoptosis.^47 , 48^ Considering the conjugated localization of BDNF-TrkB and pro-BDNF-p75 in the endothelium and due to the antagonistic physiological action between BDNF and pro-BDNF, it is important to take into account the balance between plasticity/survival and apoptosis in peripheral blood flow through the BDNF/pro-BDNF ratio.

More recently, the link between this neurotrophic and cardiovascular protection was evidenced in the study by Okada et al,^49^ conducted with conditional BDNF-knockout mice, in which BDNF expression was systemically reduced. In this study, the authors demonstrated that a mechanism mediated by the Central Nervous System is involved in the regulation of cardiac function after myocardial infarction. Ischemic insults are transmitted from the heart to the Central Nervous System through afferent cardiac fibers after the myocardial infarction, thereby increasing BDNF neuronal expression. An increase in circulating BDNF promotes the survival of cardiomyocytes and is associated with increased expression of pro-angiogenic factors. Comparatively, knockout animals had greater myocardial damage after the experimental infarction compared to wild-type mice.^49^

In this context, the Val66Met polymorphism can affect serum concentrations of BDNF and, consequently, influence the activity of tissues containing TrkB receptors, be they neurons or even peripheral tissues, such as vascular endothelial cells.

### BDNF and Cognitive Effects of Exercise

There is much evidence that physical exercise, especially aerobic exercise, has a beneficial effect on cognitive domains, particularly on executive and memory functions and reduces hippocampal atrophy in late adulthood, with BDNF being heavily involved.^11 , 50 - 57^

Epidemiological and intervention studies reinforce the idea of using physical activity as a strategy to increase neuroplasticity in pathological conditions.^58^ Several studies have shown that exercise not only causes structural changes in the brain, but also protects against aging-related cognitive decline.^57 , 59^

Physical exercise activates molecular and cellular cascades that promote neuronal plasticity and neurogenesis, inducing expression of the gene encoding BDNF.^10 , 60^ Peripheral concentrations of BDNF increase in both acute and chronic aerobic exercise, and the magnitude of this increase seems to be dependent on exercise intensity.^61^

In addition, greater cognitive benefits are obtained when the duration of the program and the exercise session are longer, individuals are older, with greater benefits for women than for men.^56^ The difference between genders regarding BDNF levels in cerebrospinal fluid in favor of women may be due to hormonal effects,^23^ since estrogen receptors are located in cells expressing BDNF and its TrkB receptor, so that estrogen regulates the expression of BDNF.^62^

Interestingly, this benefit of exercise occurs even in young adult men. This was evidenced in a cohort study of young Swedish men enlisted in military service at age 18 (n=1,221,727),^50^ in which a significant positive association was found between cardiovascular fitness and cognitive performance after adjusting for relevant confounders.

Largely, the benefits of exercise on the production of BDNF and neuronal plasticity are related to increased cerebral and muscle vascularization. In fact, in a recent review^63^ the authors have shown that the cognitive benefits of good cardiovascular fitness are related to increased cerebral circulation and angiogenesis. This important adaptation allows increased flow and upregulation of neurotrophins in the neurogenic niche of the hippocampus, a phenomenon that occurs even after acute exercise sessions.^63^

Specifically, studies on the acute and chronic effects of exercise on serum BDNF concentration still yield controversial results. For example, in a study comparing the chronic and acute effects of physical exercise on the serum concentrations of BDNF, it was demonstrated that a single exercise session was able to induce a transient increase in BDNF levels, but the same results were not achieved after a longer period of training.^64^ On the other hand, in another study where the sample was submitted to 6 months of training, a trend in an increase in serum BDNF concentration was found, in addition to an improvement in cognitive function.^65^ A similar result was found in a longitudinal study with the elderly, which resulted in an increase in the volume of hippocampal parts and, according to the authors, this fact is related to the increase in BDNF levels.^51^

These apparently controversial results may be dependent on the duration of the exercise benefits, specifically on post-exercise BDNF plasma levels, i.e., whether they occur soon after a single session of acute exercise, after a session of a regular exercise program (showing changes in BDNF release after repeated exercise sessions) or changes in resting BDNF levels after a regular exercise program.^66^ Indeed, this was evidenced in the recent meta-analysis on the effects of exercise on serum BDNF,^66^ which concluded that regular exercise intensified the effect of an exercise session on BDNF levels (Hedges’ g =0.59; *P* =0.02). However, the results indicated a lower effect of regular exercise on resting BDNF levels (Hedges’ g =0.27; *P* =0.005). There is reliable evidence from human studies indicating that each exercise episode results in a BDNF dose response and that the magnitude of this response can be increased over time through regular exercise.^66^

There is a large body of evidence that demonstrates that exercise works on several powerful neuroprotective pathways that can converge to promote continued brain health into senescence. These benefits occur either in response to acute activities or in regular practice and occur both in response to high-intensity exercises and in moderate-intensity aerobic exercises, increasing levels of circulating neurotrophic factors and neurotransmission, exerting beneficial effects on mood and cognitive functions in individuals of all ages.

### BDNF and Cardiovascular Effects of Exercise

In the cardiovascular system, BDNF is involved, at least in part, in vascular endothelial benefits. In addition, a recent study found that active older men have significantly higher plasma BDNF levels compared to their inactive peers. In this study, BDNF correlated with VO_2_max (R=0.765, p<0.001). Additionally, there was an inverse correlation between BDNF and the atherogenic index (TC / HDL), hsCRP and oxLDL. These findings demonstrate that a higher level of cardiorespiratory fitness is associated with a higher level of circulating BDNF, which in turn is related to lower cardiovascular risk.^67^

However, it is possible that polymorphisms may influence the beneficial effects of exercise. We have recently observed that peripheral vascular reactivity and serum BDNF responses to physical training are impaired by the BDNF Val66Met polymorphism, a responsiveness that is associated with serum BDNF concentrations in healthy individuals.^12^

Considering all of the above, the importance of physical exercise in promoting brain and cardiovascular health is gaining recognition, whether in the physiological condition of the brain aging process or in individuals affected by the early stages of neurodegeneration. In fact, the various animal and human studies suggest that physical activity may reduce the risk of cognitive decline, and therefore, an active lifestyle may be considered a preventive strategy for brain health deterioration, just as it occurs with cardiovascular dysfunction.

**Figure 1:** – *Acute and chronic effect of physical exercise on cardiovascular aspects related to BDNF (Adapted from Stimpson et al, 2018).*

Undoubtedly, with increasing longevity, long-term preventive approaches, with an emphasis on promoting positive health habits that delay cognitive decline and its progression, are increasingly important. It is worth remembering that in addition to modulating the internal brain environment, the regular practice of physical exercise acts directly on the cardiovascular, immune and metabolic systems, playing an essential role in a healthy lifestyle.
